# Evaluation safety and efficacy of immune checkpoint blockers (ICB) and radiotherapy combination versus ICB in non‐small cell lung cancer patients with recurrence or metastasis: A systematic review and meta‐analysis

**DOI:** 10.1002/cam4.5958

**Published:** 2023-06-16

**Authors:** Yichun Zeng, Liying Zhang, Yichen Liang, Xian Zhang, Lei Li, Maoqi Wang, Jingliang Guo, Qiuxian Li, Jin Cao, Juan J. Gu, Buhai Wang

**Affiliations:** ^1^ Medical College of Yangzhou University Yangzhou China; ^2^ Northern Jiangsu People's Hospital Yangzhou China; ^3^ Medical College of Dalian Medical University Yangzhou China

**Keywords:** combination therapy, immune checkpoint blockers, meta‐analysis, non‐small cell lung cancer, radiotherapy

## Abstract

**Background:**

Currently, immune checkpoint blockers (ICB) and radiotherapy (RT) combination therapy is broadly applied in non‐small cell lung cancer (NSCLC) patients. However, meta‐analysis about safety and efficacy of RT + ICB versus ICB has not yet been reported. To evaluate safety and efficacy of the combination therapy of ICB and RT in patients with recurrent or metastatic NSCLC and explore factors related to higher response rates, longer lifetime, and lower toxicity, meta‐analysis of previous clinical data will be presented in this article.

**Methods:**

A literature search on patients with recurrent or metastatic NSCLC treated with RT + ICB versus ICB was performed using the Cochrane Library, Embase and PubMed up to December 10, 2022. Suitable quality assessment checklists were selected corresponding to various types of research studies. Comparative and single‐arm studies were analyzed using Stata 14.0.

**Results:**

10 comparative studies and 15 arms of combination therapy were included for this meta‐analysis. RT significantly improved objective response rate (ORR), disease control rate (DCR), and overall survival (OS) and progression‐free survival (PFS) of ICB (I‐square value (*I* ^2^) = 0.00%, odds ratio (OR) 1.28, 95% confidence interval (CI) 1.09–1.49, *I* ^2^ = 0.00%, OR 1.12, 95% CI 1.00–1.25, *I* ^2^ = 42.1%, OR 0.81, 95% CI 0.72–0.92, *I* ^2^ = 34.5%, OR 0.80, and 95% CI 0.71–0.89, respectively). Toxicity between combination therapy and ICB monotherapy did not significantly differ in any grade or in ≥3 grade of tr‐AEs (*I* ^2^ = 0.00%, OR 1.05, 95% CI 0.91–1.22, *I* ^2^ = 0.00%, OR 1.46, 95% CI 0.90–2.37, respectively). Subgroup analyses based on single‐arm studies showed that applications of SRS/SBRT, PD‐1 inhibitor, and administration of ICB after RT were conducive to a better DCR, longer OS and mild adverse events (heterogeneity between groups (HBG) all *p* < 0.05).

**Conclusion:**

RT can significantly improve ORR, DCR, OS, and PFS of ICB in patients with recurrent or metastatic NSCLC without increasing toxicity. PD‐1 inhibitor following SRS/SBRT could be the best option to maximally benefit the patients.

## Graphical abstract

To evaluate safety and efficacy of combination therapy of immune checkpoint blockers (ICB) and radiotherapy (RT) in patients with advanced or recurrent non‐small cell lung cancer and explore factors related to higher response rate, longer lifetime, and lower toxicity, this article was written. The results showed that RT can significantly improve efficacy and survival of ICB and factors of the Asian population, application of traditional RT, Durvalumab as ICB combined with RT, ICB, and RT used in sequence, may enhance clinical benefits without increased toxicity.

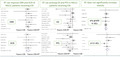

## 1 INTRODUCTION

The leading cancer‐related death is lung cancer, with 6% of 5‐year relatively survival rate,^1^ which induces a demanding need of novel regimens and therapeutic strategies to improve clinical benefit, prolong survival time, decrease treatment‐related toxicities and enhance quality of life. As immunotherapy (IT) agents, immune checkpoint blockers (ICB) (such as Pembrolizumab, Nivolumab, Durvalumab, Ipilimumab, Atezolizumab, Avelumab and Tremelizumab), which block programmed cell death‐1 (PD‐1) and PD‐1 ligand 1 (PD‐L1) or cytotoxic T‐lymphocyte‐associated antigen 4 (CTLA‐4) and eliminate cancer cells by restoring the function of immune cells. Despite some patients benefiting from treatment, improvements of response rate and survival time from IT is unsatisfactory, due to resistance to ICB.

Updated exploratory analyses from the PACIFIC Trial demonstrated the prolonged overall survival (OS) of 47.5 months and median progression‐free survival (PFS) of 17.2 months benefited patients with Durvalumab after chemoradiotherapy (CRT).^2^, ^3^ This combination including radiotherapy (RT) and IT, provided a novel perspective to overcome IT resistance and have clinical benefit. In a murine model of lung cancer, O'Donnell et al.^4^ demonstrated that the combination of IT and RT, especially stereotactic body radiotherapy (SBRT), has better anti‐tumor activity than IT alone. For patients with stage IV NSCLC, Shaverdian N et al.^5^ firstly conducted a secondary analysis comparing the efficacy and safety of RT + ICB and ICB, which showed that RT + ICB can also prolong the survival of patients with metastasis or recurrence. However, conclusions drawn from similar trials, especially prospective clinical trials, such as NCT02492568^6^ and NCT02888743^7^ are inconsistent. RT cannot significantly improve the primary resistance of ICB. In terms of sample size, the number of experimental groups in previous comparative studies usually does not exceed 100. Large multicenter randomized‐controlled clinical trials are still underway to further evaluate the clinical value of this combination. At present, there is lack of integration of existing research results on the efficacy and safety of this combination therapy.

In addition, researchers are also concerned about the influencing factors of RT and ICB combination. For example, the sequence and interval between RT and ICB are always in dispute. Some studies^8^, ^9^, ^10^ have concluded that synchronous use is more beneficial to patients, and some studies^2^, ^3^, ^7^ have shown evidence to support the use of ICB after RT. Remaining studies^11^, ^12^ concluded that the order of use does not affect the efficacy to patients. The controversy also focused on the application of new RT technology. Yang Y et al.^13^ carried out a meta‐analysis on brain RT and found that compared with conventional fractionated whole‐brain RT, the application of SRS increased survival in patients with lung cancer brain metastasis and reduced the incidence of adverse events in the nervous system. Chen D et al.^14^ compared different drugs as combined ICB and found that PD‐1 inhibitor was better than CTLA‐4 inhibitor in both efficacy and safety, which is considered to be a more suitable choice. There are still many other factors potentially related to patient efficacy, such as dosage, fractionation scheme, irradiation tumor site, whether to accept synchronous chemotherapy (CT) etc., which are rarely studied.

In view of the controversy about the efficacy and safety of adding RT on the basis of ICB, we conducted the systematic review and meta‐analysis aiming to evaluate safety and efficacy of ICB + RT vs ICB in patients with recurrent or metastatic NSCLC. Potential factors synergizing the curative effects as well as mitigating toxic side effects are further explored.

## 2 METHODS

### 2.1 Search strategy

Eligible studies were searched using the Cochrane Library, Embase, and PubMed from inception until December 10, 2022, and no language restrictions were placed. Search terms were included “NSCLC”, “RT”, “CRT”, “ICB”, “PD‐1 inhibitor”, “PD‐L1 inhibitor”, and “CTLA‐4 inhibitor”, specific drug brand/generic names (search strategies of the three database are presented in Supplement S1). Furthermore, we hand‐searched and screened websites and references of retrieved papers to avoid missing any qualified study. Our flow diagram of record selection was generated according to PRISMA.

### 2.2 Inclusion and exclusion criteria

The inclusion criteria are outlined below: (a) metastatic or recurrent NSCLC patients; (b) combination therapy using ICB and RT; (c) studies reporting efficacy and/or safety endpoints, including overall response rate (ORR), disease control rate (DCR), PFS relevant data, OS relevant data, and treatment related adverse events (tr‐AEs). No limitations were applied in publication date, region, language, study design, or sample size.

The exclusion criteria are outlined below: (a) study population concomitant with other primary malignancies, (b) studies from which relevant data was duplicated and non‐extractable, (c) studies that reported only protocols or abstracts, (d) studies that reported in special population, and (e) second analysis of previous clinical trials.

### 2.3 Quality assessment

For assessing the risk of bias, we utilized Cochrane Collaboration Risk of Bias Tool (Cochrane Handbook for Systematic Reviews of Interventions, version 5.1.0)^15^ for included randomized controlled trial (RCT) studies. Methodological index for non‐randomized studies (MINORS)^16^ was applied to evaluate single‐arm clinical trials and non‐random control trial (NRCT) studies. Cohort and case–control studies were evaluated by the Newcastle‐Ottawa Scale. JBI Critical Appraisal Checklist^17^ was used for case series.

### 2.4 Data extraction

Two investigators independently performed data extraction from the qualified studies and the third author would discuss together any differences between two investigators. Clinical features of authors, publication date of records, article type, region, study design, recruitment/case review period, follow‐up period, disease status, invention, radiation strategy, type of ICB, sample size, reported endpoints, standard of response assessment, and adverse event evaluation was recorded. Available efficacy outcomes (ORR, DCR, OS, PFS, and time‐survival rate) and safety outcomes (any grade tr‐AEs and ≥3 grade tr‐AEs) were collected. All evaluation indicators are based on the RECIST version 1.1 standard. While only survival curves were published, we extracted data from the survival curves using the Engauge Digitizer software version 10.8 and estimated the hazard ratio (HR).^18^

### 2.5 Statistical analysis

All the data were conducted with Stata 14.0, using the metan, metaprop, metaninf, and metabias (Begg's and Egger's tests) commands. The pooled effect size was measured by HR for OS and PFS, with their corresponding 95% confidence interval (CI). *p* < 0.05 was statistically significant. Fifty percent of I‐square value (*I* ^2^) was set as the cut‐off value to stratify heterogeneity into high and low levels. If the pooled effect sizes had low heterogeneity (*I* ^2^ < 50%), a fixed‐effect model was employed. If the pooled effect sizes had high heterogeneity (*I* ^2^ ≥ 50%), we used a random‐effects model and further analyzed potential factors using subgroup analysis, meta‐regression, and sensitivity analysis.

## 3 RESULTS

### 3.1 Study characteristics

**FIGURE 1:**
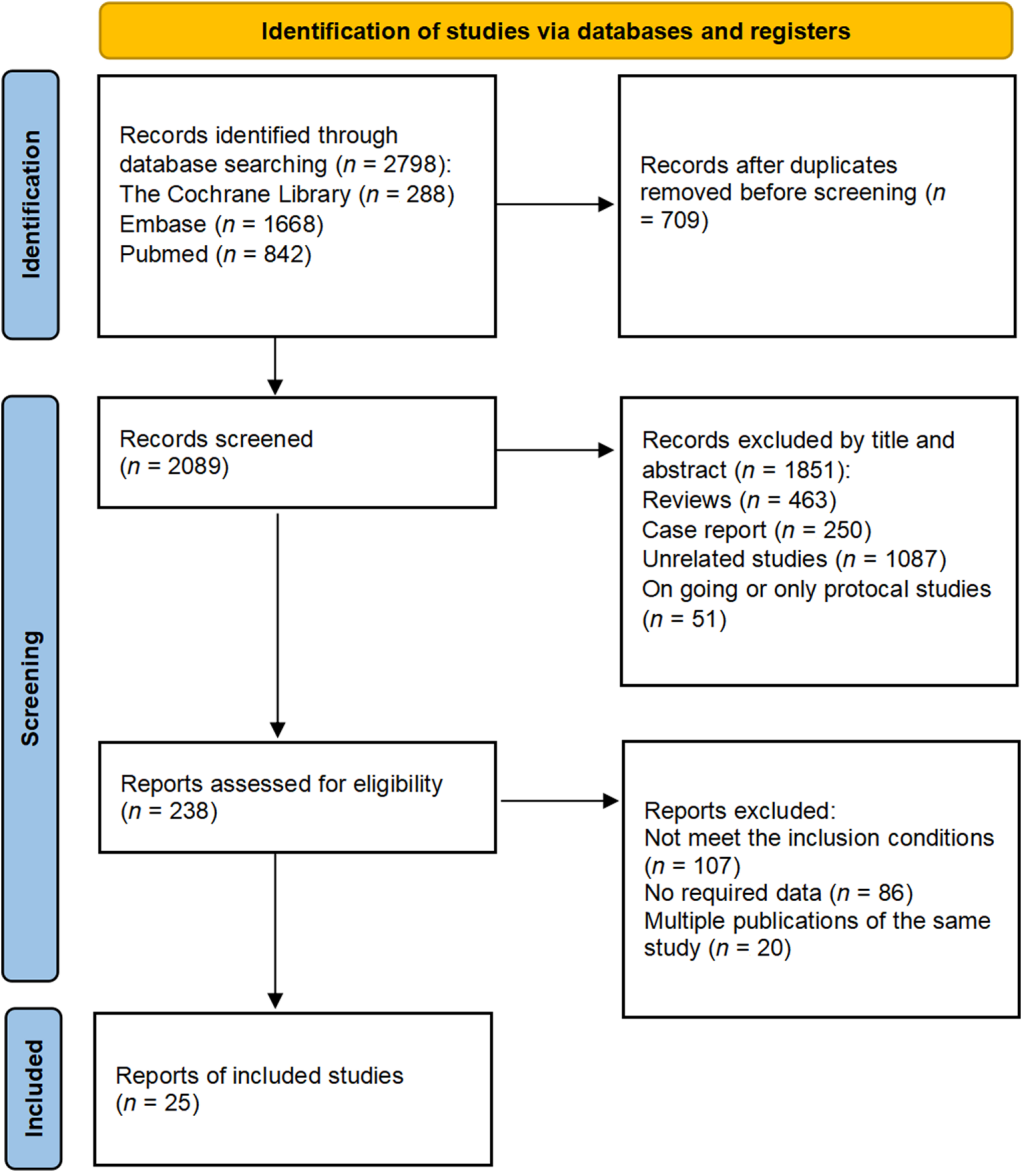
Flow diagram of the identification of eligible studies.

The flowchart of the literature search is presented in Figure 1. We retrieved 2798 records from three databases, of which 709 duplicated records were excluded. After a series of filters, eventually, 25 journal articles^6^, ^7^, ^8^, ^9^, ^10^, ^11^, ^12^, ^19^, ^20^, ^21^, ^22^, ^23^, ^24^, ^25^, ^26^, ^27^, ^28^, ^29^, ^30^, ^31^, ^32^, ^33^, ^34^, ^35^, ^36^ were included for the present meta‐analysis. In some articles, two or more arms of combined treatment of ICB + RT in articles were analyzed separately in our meta‐analysis. The detailed information of comparative studies of ICB plus RT vs ICB is presented in Table 1. Overall, 11 articles with 1821 NSCLC patients were included in this meta‐analysis. We presented eligible articles containing ICB + RT arms in Table 2, in which 14 arms including 551 NSCLC patients treated with ICB + RT were included.

**TABLE 1:** Characteristics of comparative studies.

Study	Country	Study design	Recruitment/case review period	Follow‐up time [range] months	ICB	Type of radiotherapy	Site of radiotherapy	With concurrent chemotherapy	Sample size	Sequence between ICB and radiotherapy
Fiorica F, 2018	Italy	Retrospective	NA	7.3 [2.3–12.5]	Nivolumab	HRT	Multi	No	15	c/s
					Nivolumab	—	—	No	20	—
Guo T, 2022	China	Retrospective	2017.06–2020.09	13.2 [0.8–44.4]	PD‐1/PD‐L1 blockade	Multi	Brain	Some	26	c/s
					PD‐1/PD‐L1 blockade	—	—	Some	84	—
Hosokawa S, 2020	Japan	Retrospective	2015.12–2018.05	NA	PD‐1/PD‐L1 blockade	Any RT	Chest	Some	139	Sequential
					PD‐1/PD‐L1 blockade	—	—	Some	392	—
Ratbayake G, 2019	Britain	Retrospective	2015.07–2016.12	15	Nivolumab	Any RT	Multi	No	65	c/s
					Nivolumab	—	—	No	20	—
Schoenfeld JD,2022	USA	Prospective	2017.08–2019.04	12.4 [7.8–15.1]	Durvalumab+Tremelimumab	Any RT	Multi	No	26	Concurrent
					Durvalumab+Tremelimumab	—	—	No	52	—
Samuel E, 2020	Australia	Retrospective	2016.01–2019.05	19.4	NA	Any RT	Multi	No	102	Concurrent
					NA	—	—	No	167	—
Theelen WSME, 2019	Netherland	Prospective	2015.07–2018.03	23.6 [0.1–34.4]	Pembrolizumab	SBRT	Multi	No	36	Sequential
					Pembrolizumab	—	—	No	40	—
Welsh J, 2020	USA	Prospective	2015.09–2018.08	20.4	Pembrolizumab	SBRT/tRT	Lung/liver	No	40	Concurrent
					Pembrolizumab	—	—	No	40	—
Yamaguchi O, 2019	Japan	Retrospective	2016.02–2017.12	NA	PD‐1 blockade	Any RT	Multi	No	66	c/s
					PD‐1 blockade	—	—	No	58	—
Zhang Q, 2022	China	Retrospective	2019.01–2021.12	NA	Camrelizumab	tRT	Brain	No	67	NA
					Camrelizumab	—	—	No	85	—
Zhou ZC, 2022	China	Retrospective	2017.06–2020.12	28	PD‐1/PD‐L1 blockade	Any RT	Multi	Some	107	c/s
					PD‐1/PD‐L1 blockade	—	—	Some	214	—

Abbreviations: any RT, any kind of radiotherapy; c/s, concurrent or sequential; cRT, conventional radiotherapy; HRT, hyperfractionated radiotherapy; NA, not achieved; SBRT, stereotactic body radiation therapy; TRT, thoracic radiotherapy.

**TABLE 2:** Characteristics of single‐arm studies.

Study	Country	Study design	Recruitment/ case review period	Follow‐up time [range] (months)	Treatment line	ICB	Type of radiotherapy	Site of radiotherapy	Concurrent chemotherapy	Sample size	Sequence between ICB and RT
Abdulhaleem M, 2022	USA	Retrospective	2014.10–2019.09	NA	NA	Nivolumab/Atezolizumab/ Pembrolizumab/Durvalumab	SRS	Brain	No	80	c/s
Amino Y, 2019	Japan	Retrospective	2013.03–2018.04	13.5 [0.0–57.0]	2	Nivolumab/Pembrolizumab	NA	Chest	Yes	20	c/s
Bassanelli M, 2022	Italy	Retrospective	NA	NA	≥2	Nivolumab	tRT	Multi	NA	95	c/s
Bestvina CM, 2022	USA	Prospective	2017.09–2019.11	17	1	Ipilimumab, Nivolumab	SBRT	Multi	Some	37	c/s
Horndalsveen H, 2022	Norway	Prospective	2018.09–2020.02	26.5[17.6–35.5]	≥2	Atezolizumab	SBRT	Chest	No	21	Concurrent
Hubbling HG, 2018	USA	Retrospective	2013.08–2016.09	NA	1/2	anti‐PD‐1 antibodies	Any RT	Brain	No	50	c/s
Lesueur P, 2018	France	Retrospective	NA	NA	Multi	Nivolumab	Any RT	Multi	No	104	c/s
Mattes MD, 2021	USA	Prospective	2017.03–2019.04	14.0 [5.4–23.5]	NA	anti‐PD‐1 antibodies	SBRT	Multi	No	35	c/s
Miyamoto S, 2018	Japan	Prospective	2016.09–2017.09	NA	≥2	Nivolumab	SBRT	Chest	No	6	Sequential
Porte J, 2022	France	Retrospective	2015.02–2019.12	22.5[2.7–47.3]	1/2	Nivolumab/Atezolizumab/ Pembrolizumab	SRT	Brain	No	51	c/s
Qin A, 2019	USA	Prospective	2015.10–2017.02	NA	Multi	Atezolizumab	HIGRT	Multi	No	12	Concurrent
Schapira E, 2018	USA	Retrospective	2012–2017	14.3 [5.1–53.1]	NA	Nivolumab/Atezolizumab/ Pembrolizumab	SRS	Brain	No	37	c/s
Singh C, 2019	USA	Retrospective	2006.01–2016.09	12 [6–132]	NA	anti‐PD‐1 antibodies	SRS	Brain	No	39	NA
Tjong MC, 2022	Canada	Retrospective	2014.06–2019.07	9.7[3.1–20.3]	Multi	Nivolumab/Atezolizumab/ Pembrolizumab/Durvalumab	Any RT	Multi	Yes	64	c/s

Abbreviations: any RT, any kind of radiotherapy; c/s, concurrent or sequential; tRT, traditional radiotherapy; HIGRT, hypofractionated Image‐guided Radiotherapy; HRT, hyperfractionated radiotherapy; NA, not achieved; SBRT, stereotactic body radiation therapy; SRS, stereotactic radiosurgery.

### 3.2 Quality assessment

In general, the quality items of 3 RCTs, 2 NRCTs, 12 case control studies, 1 single‐arm studies, and 7 case series were well reported. For three RCTs, we used Cochrane Collaboration's tool to assess risk of bias and evaluated the overall risk of bias as low risk (Table S1 presented in Supplement S2). For two NRCTs and one single‐arm studies, we scored them from 14 to 15 points using MINORS index (Table S2 presented in Supplement S2), which were acceptable for the present meta‐analyses. Case control studies with NOS scores greater than 6 were all eligible (Table S3 presented in Supplement S2). Seven case series were included after they were evaluated by the JBI Critical Appraisal Checklist scored 14–17 points (Table S4 presented in Supplement S2).

### 3.3 Efficacy and safety in comparative studies

**FIGURE 2:**
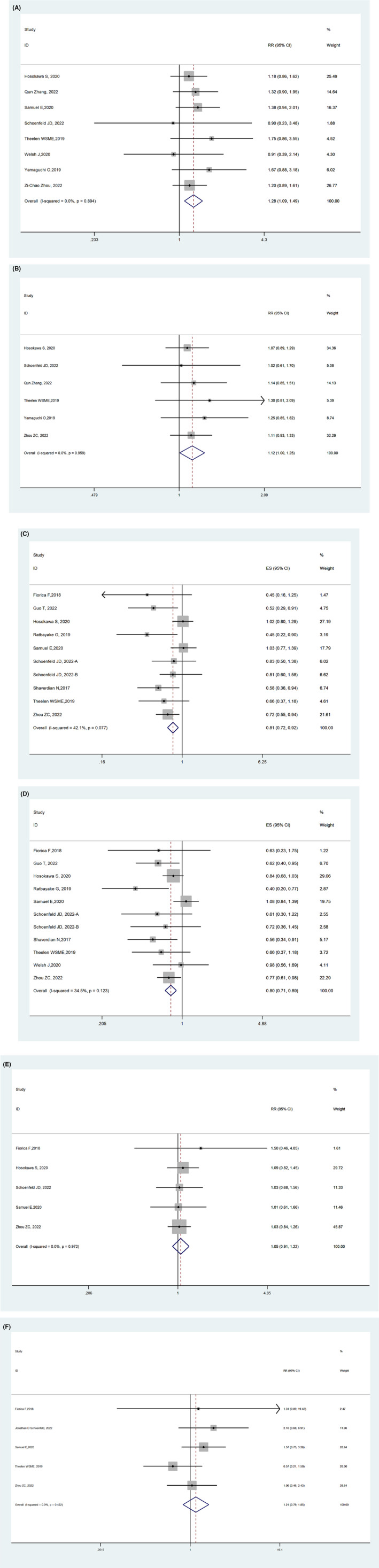
Pooled results of efficacy and safety in comparative studies. (A) ORR in in comparative studies; (B) DCR in comparative studies; (C) OS in comparative studies; (D) PFS in comparative studies; (E) incidence of any grade treatment‐related advert events in comparative studies; (F) incidence of ≥3 grade treatment‐related advert events in comparative studies.

Results of the meta‐analysis in 10 studies^6^, ^7^, ^19^, ^20^, ^21^, ^22^, ^23^, ^24^, ^25^, ^26^, ^27^, ^28^, ^29^, ^30^, ^31^, ^32^, ^33^, ^34^, ^35^, ^36^ comparing ICB plus RT with ICB were performed in Figure 2, ICB combined with RT was better than without it both in short‐term and long‐term efficacy without increased toxicity. ORR, DCR, OS, and PFS were significantly inferior in patients with combination therapy (*I* ^2^ = 0.00%, OR 1.31, 95% CI 1.13–1.52, *I* ^2^ = 0.00%, OR 1.13, 95% CI 1.02–1.26, *I* ^2^ = 42.1%, OR 0.81, 95% CI 0.72–0.92, *I* ^2^ = 34.5%, OR 0.80, and 95% CI 0.71–0.89, respectively). In studies available for, results of the meta‐analysis showed toxicity between combination therapy and ICB monotherapy did not significantly differ in any grade or in ≥3 grade of tr‐AEs (*I* ^2^ = 0.00%, OR 1.05, 95% CI 0.91–1.22, *I* ^2^ = 0.00%, OR 1.46, and 95% CI 0.90–2.37, respectively). All the literature was examined using the Begg's and Egger's tests, and there was no potential publication bias.

### 3.4 Subgroup analyses based on single‐arm study

We defined the arm of ICB combined with RT as a single‐arm test for analysis, for articles without our comparison content in their control group. Since most studies had no control group, we conducted a single‐arm meta‐analysis of available outcomes in included studies. The number of single‐arm studies and heterogeneity was relatively large, and heterogeneous. Subgroup analysis was used to further explore efficacy and safety. Considering representativeness and comprehensiveness of results, we selected DCR, 2‐year OS rate and ≥3 grade tr‐AEs as evaluation factors of efficacy, survival time and therapeutic safety. Subgroup analyses based on region, type of RT, and type of ICB, whether combined with concurrent CT, disease status, sequence between radiation, and ICB indicated in Table 3. For patients with metastasis or recurrence, more attention focused on whether treatment strategies can prevent disease progression. IT emphasizes disease control and maintenance, compared to CT. Therefore, DCR was chosen to be the clinical evaluation index, instead of ORR. Population of Asia, ICB + ICB and administration of ICB after RT was conducive to better DCR (heterogeneity between groups (HBG) all *p* < 0.005). Different from meta‐analysis in comparative studies, which can directly compare PFS and OS of patients, we can only analyze binary variables in single‐arm studies, that is, time survival rate. Considering follow‐up periods of trials, we selected the 2‐years as the time point for OS subgroup analysis. The analysis of a 2‐year OS rate could be concluded that SRS/SBRT, PD‐L1 inhibitor, combined with concurrent CT, and sequential treatment between ICB and RT were related factors affecting the survival period (HBG *p* < 0.005, *p* < 0.005, *p* < 0.005, *p* < 0.005, and *p* < 0.05, respectively). Grade 3 or greater tr‐AEs associated with patients' quality of life and adherence to medication were selected as a representative indicator of therapeutic safety to explore the possible related factors. According to the results in Asian populations, PD‐1 inhibitor and sequential treatment were safety factors. On the contrary in North American populations, ICB + ICB and concurrent ICB + RT were related factors to obtain severe adverse events. All the literature were examined using the Begg's and Egger's tests, and there was no potential publication bias.

**TABLE 3:** Subgroup analyses of single‐arm studies.

	DCR				2‐year OS rate				≥G3 AEs rate			
Group	No. of studies	ES (95% CI)	*I* ^2^ (%)	HBG (p)	No. of studies	ES (95% CI)	*I* ^2^ (%)	HBG (p)	No. of studies	ES (95% CI)	*I* ^2^ (%)	HBG (p)
Total	12	0.68 (0.56, 0.80)	88.38		15	0.42 (0.33, 0.51)	84.85		13	0.16 (0.10,0.23)	80.81	
Region												
Asia	5	0.85 (0.81, 0.88)	87.9	**	4	0.50 (0.45, 0.55)	85.7	**	2	0.08 (0.03, 0.13)	0.0	*
Europe	3	0.67 (0.58, 0.76)	0.0		4	0.31 (0.25, 0.37)	73.6		4	0.10 (0.05, 0.14)	0.0	
North America	3	0.65 (0.56, 0.75)	31.8		6	0.49 (0.43, 0.54)	79.8		6	0.15 (0.12, 0.19)	95.1	
Oceania	1	0.38 (0.29, 0.48)	—		1	0.20 (0.12, 0.27)	—		1	0.13 (0.06, 0.19)	—	
Type of RT												
tRT	3	0.75 (0.68, 0.83)	63.4	—	2	0.26 (0.18, 0.34)	0.0	**	1	0.24 (0.07, 0.41)	—	—
HRT	—	—	—		2	0.22 (0.11, 0.32)	0.0		1	0.07 (−0.08, 0.21)	—	
SBRT	4	0.68 (0.60, 0.77)	0.0		3	0.48 (0.40, 0.57)	0.0		5	0.07 (0.03, 0.11)	58.9	
SRS	—	—	—		3	0.45 (0.37, 0.52)	85.3		—	—	—	
Type of ICI												
ICB + ICB	2	0.61 (0.51, 0.71)	0.0	**	2	0.46 (0.39, 0.53)	—	**	2	0.38 (0.29, 0.48)	84.6	**
PD‐1 inhibitor	6	0.72 (0.66, 0.77)	14.0		4	0.28 (0.22, 0.34)	60.4		6	0.13 (0.09, 0.16)	52.1	
PD‐L1 inhibitor	3	0.43 (0.35, 0.51)	40.1		1	0.25 (0.03, 0.47)	—		0	—		
With concurrent chemotherapy												
Yes	1	0.65 (0.46, 0.84)	—	—	2	0.50 (0.40, 0.60)	91.1	**	0	—	9	—
No	8	0.63 (0.58, 0.68)	85.0		8	0.33 (0.29, 0.38)	84.5		11	0.13 (0.10, 0.16)	68.4	
Sequence between ICI and RT												
Concurrent	2	0.40 (0.31, 0.48)	0.0	**	3	0.24 (0.16, 0.31)	69.7	*	4	0.18 (0.13, 0.23)	60.2	—
Sequential	4	0.68 (0.62, 0.74)	0.0		3	0.36 (0.30, 0.43)	88.2		2	0.14 (0.04, 0.25)	0.0	

*Note*: Considerable heterogeneity observed in one or more sub‐groups, test for heterogeneity between sub‐groups likely to be invalid. *p < 0.05; **p < 0.005; NA, *p* ≥ 0.05.

Abbreviations: ≥G3 AEs, treatment‐related ≥3 grade advert events; tRT, traditional radiotherapy; DCR, disease control rate; HBG, heterogeneity between groups; HRT, hyperfractionated radiotherapy; SBRT, stereotactic body radiation therapy; SRS, stereotactic radiosurgery.

Analyzing short‐term efficacy, long‐term efficacy, and safety together in the asian population it was observed that ICB and RT used in sequence had favorable efficacy and lower toxicity. Application of SRS/SBRT could improve OS and did not show serious toxicity compared to RT techniques. As for ICB selection, our research results showed that, although the patients receiving PD‐1/PD‐L1 inhibitor + CTLA‐4 inhibitor could increase the 2‐year OS rate and may withstand more serious toxicity. Therefore, it was determined which ICB was more suitable for combined RT and ICB therapy from a horizontal comparison. Regarding CT, current data is difficult to determine its use value.

## 4 DISCUSSION

It is known that ICB + RT is one of the most effective therapy strategies among the different therapeutic modalities. Preclinical models revealed unconventional fractionation RT techniques especially SBRT combined with ICB was the most effective combination.^37^ In terms of sequence of RT and IT, administration of CTLA‐4 inhibitors can induce anti‐tumor immunity both before and after SBRT,^4^ however PD‐1/PD‐L1 inhibitors are ideally administrated during SBRT and need to continue for some days.^38^ International multi‐center research^2^, ^3^ has confirmed that CRT sequential ICB can significantly improve the survival time of patients with stage III lung cancer. The controversy lies in whether RT as local treatment has a positive effect on disease control and maintenance of patients based on systematic ICB treatment of patients with NSCLC in stage IV. Currently, there are few large‐scale multi‐center RCT results to confirm clinical data consistent with preclinical results. At present, scholars have different opinions on whether the sequence between RT and ICB, interval time, new RT technology, and other potential factors can affect efficacy and safety of combination treatment. Therefore, our study provides a systematic review and meta‐analysis of available clinical data regarding the usage of radioimmunotherapy in NSCLC.

Efficacy outcomes of our meta‐analysis proved that ICB combined with RT was superior to single therapy of ICB in ORR, DCR, OS, and PFS, which was not proved conclusively in a previous study. From meta‐analyses of Geng Y et al.^39^ and Fiorica F et al.,^40^ conclusions are similar, that IT plus RT only improved ORR, but did not confirm the prolongation of survival time in patients. Due to the publication of the new clinical research results, our meta‐analysis integrated the latest comprehensive data and confirmed that the combination therapy played a role in improving the short‐term and long‐term efficacy in patients with advanced or recurrent NSCLC. Meta‐analysis results of safety, consistent with points of those studies, indicated that additional RT to ICB did not increase the incidence of any grade tr‐AEs or ≥3 grade tr‐AEs. To our knowledge, it is the first meta‐analysis report of this combination therapy, confirming prolonged OS and PFS. Mo et al. study^41^ has proved that the addition of CT on the basis of ICB prolongs PFS and OS of patients but increases the incidence of ≥3 grade tr‐AEs. There are several reasons why previous meta‐analysis failing to demonstrate this. One possible cause is that new clinical data was published in the last 2 years, which were first enrolled in survival time meta‐analysis. Second and more importantly, our study extracted time‐to‐event data of Kaplan–Meier's survival curves. Adoption of this method generating HR from published time‐to‐event data enabled limited original clinical data be fully utilized.^18^

To select the most suitable treatment population, our research focused on identifying the factors influencing the efficacy and safety of the combination therapy. Based on the outcomes from subgroup analyses of combined effect in single‐arm studies, we concluded that in the Asian population, application of PD‐1 inhibitors and administration of ICB after RT are favorable for efficacy and lower toxicity. Since treatment strategy combined with concurrent CT and disease status of stage III improved DCR and OS but aggravated toxicity, it should be applied cautiously. However, from comparative studies included in this review, we observed that most of their experimental design was composed of clinical features without concurrent CT, ICB using anti‐PD‐1 inhibitor Nivolumab/Pembrolizumab, and treatment in stage IV/PD NSCLC patients, which were certified as potential disadvantages. In turn, if favorable factors are met in the experimental design, the efficacy would be further improved. Considering the complexity of efficacy and safety in different disease statuses, more comparative studies are needed.

RT‐related factors, including dosage, fractionation scheme, site, and novel techniques, are the focus of our study potentially affecting the efficacy and toxicity of the combined therapy. In terms of metastases, the most frequent locations are lung, mediastinum, liver, and brain. Among patients with stage IV lung cancer, brain metastasis are the most studied. The effect of RT based on ICB is confirmed, which can control local lesions and prolong the survival of patients. SRS technology is popular among patients with brain metastasis, although it will increase adverse events of nervous systems. Liver metastasis is one of the most common metastatic sites, with the worst prognosis, the shortest survival period, and poor efficacy of PD‐1 inhibitors. The existing studies have not yet taken the population with lung cancer and liver metastasis as the research object alone, so it is impossible to know whether ICB + RT is effective for the population with liver metastasis. In terms of dosage and fractionation scheme, most of the studies included in this paper used unconventional fractionation, and the fractionation dose exceeds 5gy. Based on preclinical studies, RT and ICB have a synergistic effect. Some experts^6^ divided the patients into high and low dose groups and found that hypo fractionated RT was related to higher ORR.

Besides RT related factors, we also analyzed the impact of other factors on treatment strategies. One is the sequence and interval time between ICB and RT. Updated exploratory analyses from the PACIFIC Trial^2^, ^3^ showed the prolonged median OS and median PFS benefited stage III patients with Durvalumab after CRT. However, the sequence between ICB and RT in NSCLC patients with metastasis or recurrence remains contested, and in some studies,^8^, ^9^, ^10^ using concurrent treatment with RT and ICB benefited patients. According to our results, administration of ICB after RT achieved better efficacy without an increase in incidence of ≥3 grade tr‐AEs. The choice of ICB is also one of the key factors. Chen D et al.^14^ carried out a secondary analysis on the comparison of Ipilimumab+SBRT and Pembrolizumab+SBRT, and found that CTLA‐4 was not as effective as Pembrolizumab and brought strong adverse reactions to patients. Their evidence supports our subgroup study that the use of PD‐1/PD‐L1 inhibitor combined with CTLA‐4 significantly increased the incidence of ≥3 grade tr‐AEs in patients. CT, which has been demonstrated to improve the primary resistance of ICB, is the standard treatment scheme for patients with unresectable stage III NSCLC. In the treatment of patients with stage IV NSCLC, it is worth exploring whether to use concurrent CT, which is also one of the factors affecting the efficacy of combined therapy. According to our data, CT can prolong the survival period of patients, and evidence reported by Geng et al.^39^ support this conclusion.

The present review does have several limitations. First, most of the included studies were retrospective with lack of a control group. Besides, in comparative studies, some of them designed RT or CRT as control group were not in line with the purpose of our research. Only 2 RCTs reported results for comparison. Second, the single‐arm analysis only can combine effects from the data of binary variables not for measurement data and contributed to significant heterogeneity. Therefore, the reference value of the subgroup results analysis is limited. Third, although we had carried out subgroup analyses for baseline prognostic characteristics and various therapy regimens. Current data cannot satisfy the needs of such analyses of other underlying factors, such as age, ECOG performance, histological types, tumor PD‐L1 expression, NSCLC‐associated genes driver mutation status, the dose of RT, different irradiated sites, etc. However, we still found meaningful results which may provide reference to clinicians. In the near future, we are looking forward to analyze more large‐scale multi‐center RCTs to determine increase curative effects from this combination, which can help to select a suitable ICB and sequence between RT and ICB and to screen out suitable patients.

## 5 CONCLUSION

From analyses of completed comparative clinical studies, ICB combined with RT showed superior ORR, DCR, OS, and PFS than ICB without RT, without an increase in any grade or ≥3 grade tr‐AEs. According to subgroup analysis, comprehensively, PD‐1 inhibitor following SRS/SBRT could be the best option to maximally benefit patients. Moreover, on‐going large‐scale RCTs are expected to further confirm this notion.

## AUTHOR CONTRIBUTIONS

**Yichun Zeng:** Conceptualization (lead); data curation (lead); formal analysis (lead); methodology (lead); writing – original draft (lead). **Liying Zhang:** Conceptualization (lead); data curation (lead); methodology (lead); resources (lead). **Yichen Liang:** Supervision (equal); writing – review and editing (equal). **Xian Zhang:** Supervision (equal); validation (equal); writing – review and editing (equal). **Lei Li:** Supervision (equal); writing – review and editing (equal). **Maoqi Wang:** Supervision (equal); validation (equal); writing – review and editing (equal). **Jingliang Guo:** Supervision (equal); validation (equal); writing – review and editing (equal). **Qiuxian Li:** Supervision (equal); writing – review and editing (equal). **Jing Cao:** Supervision (equal); writing – review and editing (equal). **Juan J Gu:** Supervision (lead); writing – review and editing (lead). **Buhai Wang:** Funding acquisition (lead); project administration (lead); supervision (lead); writing – review and editing (lead).

## FUNDING INFORMATION

This study was supported by the science and technology bureau fund of Yangzhou City (No. YZ2019057).

## CONFLICT OF INTEREST STATEMENT

The authors declared that they had no conflict of interests.

## Supporting information


Supplementary Information S1.

Supplementary Information S2.
Table S1.
Table S2.
Table S3.
Table S4.

Supplementary information S3.

## Data Availability

All data generated and analyzed during this study are included in this article.
