# Supplementary material for: Evaluation safety and efficacy of immune checkpoint blockers (ICB) and radiotherapy combination versus ICB in non‐small cell lung cancer patients with recurrence or metastasis: A systematic review and meta‐analysis
Source: Cancer Med. 2023 Jun 16;12(13):13928–41. doi: 10.1002/cam4.5958 (PMC10358264; doi:10.1002/cam4.5958)
Supplement: Supplementary file 1 — Supplementary Information S1. [file CAM4-12-13928-s001.doc]

Supplement information 1. Search strategy

pubmed

(("radiotherapies*"[Title/Abstract] OR "radiation therapy*"[Title/Abstract] OR "radiation therapies*"[Title/Abstract] OR "therapies radiation*"[Title/Abstract] OR "therapy radiation*"[Title/Abstract] OR "radiation treatment*"[Title/Abstract] OR "radiation treatments*"[Title/Abstract] OR "treatment radiation*"[Title/Abstract] OR "radiotherapy targeted*"[Title/Abstract] OR "radiotherapies targeted*"[Title/Abstract] OR "targeted radiotherapies*"[Title/Abstract] OR "targeted radiotherapy*"[Title/Abstract] OR "targeted radiation therapy*"[Title/Abstract] OR (("radiate"[All Fields] OR "radiated"[All Fields] OR "radiates"[All Fields] OR "radiating"[All Fields] OR "Radiation"[MeSH Terms] OR "Radiation"[All Fields] OR "electromagnetic radiation"[MeSH Terms] OR ("electromagnetic"[All Fields] AND "Radiation"[All Fields]) OR "electromagnetic radiation"[All Fields] OR "radiations"[All Fields] OR "radiation s"[All Fields] OR "radiator"[All Fields] OR "radiators"[All Fields]) AND "therapies targeted*"[Title/Abstract]) OR "targeted radiation therapies*"[Title/Abstract] OR (("therapeutics"[MeSH Terms] OR "therapeutics"[All Fields] OR "Therapies"[All Fields] OR "Therapy"[MeSH Subheading] OR "Therapy"[All Fields] OR "therapy s"[All Fields] OR "therapys"[All Fields]) AND "targeted radiation*"[Title/Abstract]) OR "therapy targeted radiation*"[Title/Abstract] OR ("Radiotherapy"[MeSH Terms] OR "Radiotherapy"[All Fields] OR ("Radiation"[All Fields] AND "Therapy"[All Fields] AND "Targeted"[All Fields]) OR "radiation therapy targeted"[All Fields])) AND ("carcinoma non small cell lung*"[Title/Abstract] OR (("carcinoma, non small cell lung"[MeSH Terms] OR ("Carcinoma"[All Fields] AND "Non-Small-Cell"[All Fields] AND "Lung"[All Fields]) OR "non-small-cell lung carcinoma"[All Fields] OR ("Carcinoma"[All Fields] AND "Non"[All Fields] AND "Small"[All Fields] AND "Cell"[All Fields] AND "Lung"[All Fields]) OR "carcinoma non small cell lung"[All Fields]) AND "carcinomas non small cell lung*"[Title/Abstract]) OR "lung carcinoma non small cell*"[Title/Abstract] OR "lung carcinomas non small cell*"[Title/Abstract] OR "non small cell lung carcinomas*"[Title/Abstract] OR "non small cell lung carcinoma*"[Title/Abstract] OR "non small cell lung carcinoma*"[Title/Abstract] OR (("carcinoma, non small cell lung"[MeSH Terms] OR ("Carcinoma"[All Fields] AND "Non-Small-Cell"[All Fields] AND "Lung"[All Fields]) OR "non-small-cell lung carcinoma"[All Fields] OR ("Non"[All Fields] AND "Small"[All Fields] AND "Cell"[All Fields] AND "Lung"[All Fields] AND "Carcinoma"[All Fields]) OR "non small cell lung carcinoma"[All Fields]) AND "nonsmall cell lung cancer*"[Title/Abstract]) OR "carcinoma non small cell lung*"[Title/Abstract] OR "non small cell lung cancer*"[Title/Abstract] OR "carcinomas non small cell lung*"[Title/Abstract] OR "lung carcinoma non small cell*"[Title/Abstract] OR "lung carcinomas non small cell*"[Title/Abstract] OR "non small cell lung carcinomas*"[Title/Abstract] OR "non small cell lung carcinoma*"[Title/Abstract] OR "non small cell lung carcinoma*"[Title/Abstract] OR (("carcinoma, non small cell lung"[MeSH Terms] OR ("Carcinoma"[All Fields] AND "Non-Small-Cell"[All Fields] AND "Lung"[All Fields]) OR "non-small-cell lung carcinoma"[All Fields] OR ("Non"[All Fields] AND "Small"[All Fields] AND "Cell"[All Fields] AND "Lung"[All Fields] AND "Carcinoma"[All Fields]) OR "non small cell lung carcinoma"[All Fields]) AND "nonsmall cell lung cancer*"[Title/Abstract]) OR "carcinoma non small cell lung*"[Title/Abstract] OR ("carcinoma, non small cell lung"[MeSH Terms] OR ("Carcinoma"[All Fields] AND "Non-Small-Cell"[All Fields] AND "Lung"[All Fields]) OR "non-small-cell lung carcinoma"[All Fields] OR ("Non"[All Fields] AND "Small"[All Fields] AND "Cell"[All Fields] AND "Lung"[All Fields] AND "cancer"[All Fields]) OR "non small cell lung cancer"[All Fields])) AND ("checkpoint inhibitors immune*"[Title/Abstract] OR (("Immune Checkpoint Inhibitors"[Pharmacological Action] OR "Immune Checkpoint Inhibitors"[MeSH Terms] OR ("Immune"[All Fields] AND "Checkpoint"[All Fields] AND "Inhibitors"[All Fields]) OR "Immune Checkpoint Inhibitors"[All Fields] OR ("Immune"[All Fields] AND "Checkpoint"[All Fields] AND "Inhibitor"[All Fields]) OR "immune checkpoint inhibitor"[All Fields]) AND "checkpoint inhibitor immune*"[Title/Abstract]) OR "immune checkpoint blockers*"[Title/Abstract] OR "checkpoint blockers immune*"[Title/Abstract] OR "immune checkpoint blockade*"[Title/Abstract] OR "checkpoint blockade immune*"[Title/Abstract] OR "immune checkpoint inhibition*"[Title/Abstract] OR "checkpoint inhibition immune*"[Title/Abstract] OR "pd l1 inhibitors*"[Title/Abstract] OR "pd l1 inhibitors*"[Title/Abstract] OR "pd l1 inhibitor*"[Title/Abstract] OR "pd l1 inhibitor*"[Title/Abstract] OR "programmed death ligand 1 inhibitors*"[Title/Abstract] OR "programmed death ligand 1 inhibitors*"[Title/Abstract] OR "pd 1 pd l1 blockade*"[Title/Abstract] OR "blockade pd 1 pd l1*"[Title/Abstract] OR "pd 1 pd l1 blockade*"[Title/Abstract] OR "ctla 4 inhibitors*"[Title/Abstract] OR "ctla 4 inhibitors*"[Title/Abstract] OR "ctla 4 inhibitor*"[Title/Abstract] OR "ctla 4 inhibitor*"[Title/Abstract] OR "cytotoxic t lymphocyte associated protein 4 inhibitors*"[Title/Abstract] OR "cytotoxic t lymphocyte associated protein 4 inhibitors*"[Title/Abstract] OR "cytotoxic t lymphocyte associated protein 4 inhibitor*"[Title/Abstract] OR "cytotoxic t lymphocyte associated protein 4 inhibitor*"[Title/Abstract] OR "pd 1 inhibitors*"[Title/Abstract] OR "pd 1 inhibitors*"[Title/Abstract] OR "pd 1 inhibitor*"[Title/Abstract] OR "inhibitor pd 1*"[Title/Abstract] OR "pd 1 inhibitor*"[Title/Abstract] OR "programmed cell death protein 1 inhibitor*"[Title/Abstract] OR ("Immune Checkpoint Inhibitors"[Pharmacological Action] OR "Immune Checkpoint Inhibitors"[MeSH Terms] OR ("Immune"[All Fields] AND "Checkpoint"[All Fields] AND "Inhibitors"[All Fields]) OR "Immune Checkpoint Inhibitors"[All Fields] OR "programmed cell death protein 1 inhibitors"[All Fields]) OR "Immune Checkpoint Inhibitors"[MeSH Terms])) OR (("toripalimab"[Supplementary Concept] OR "toripalimab*"[Title/Abstract] OR ("avelumab"[Supplementary Concept] OR "avelumab*"[Title/Abstract] OR "msb0010682*"[Title/Abstract] OR "bavencio*"[Title/Abstract] OR "msb0010718c*"[Title/Abstract] OR "msb 0010718c*"[Title/Abstract]) OR ("Ipilimumab"[MeSH Terms] OR "ipilimumab*"[Title/Abstract] OR "anti ctla 4 mab ipilimumab*"[Title/Abstract] OR "anti ctla 4 mab ipilimumab*"[Title/Abstract] OR "ipilimumab anti ctla 4 mab*"[Title/Abstract] OR "yervoy*"[Title/Abstract] OR "mdx 010*"[Title/Abstract] OR "mdx010*"[Title/Abstract] OR "mdx 010*"[Title/Abstract] OR "mdx ctla 4*"[Title/Abstract] OR "mdx ctla 4*"[Title/Abstract]) OR ("tremelimumab"[Supplementary Concept] OR "tremelimumab*"[Title/Abstract] OR "ticilimumab*"[Title/Abstract] OR "cp 675*"[Title/Abstract] OR ("CP675"[All Fields] AND "cpd"[Title/Abstract]) OR "cp 675*"[Title/Abstract] OR "cp 675 206*"[Title/Abstract] OR "cp 675206*"[Title/Abstract] OR "cp675206*"[Title/Abstract] OR "cp 675206*"[Title/Abstract]) OR ("cemiplimab"[Supplementary Concept] OR "cemiplimab*"[Title/Abstract] OR "regn2810*"[Title/Abstract]) OR ("durvalumab"[Supplementary Concept] OR "durvalumab*"[Title/Abstract] OR "medi4736*"[Title/Abstract] OR "medi 4736*"[Title/Abstract] OR "imfinzi*"[Title/Abstract]) OR ("Nivolumab"[MeSH Terms] OR "nivolumab*"[Title/Abstract] OR "opdivo*"[Title/Abstract] OR "ono 4538*"[Title/Abstract] OR "ono 4538*"[Title/Abstract] OR "ono4538*"[Title/Abstract] OR "mdx 1106*"[Title/Abstract] OR "mdx 1106*"[Title/Abstract] OR "mdx1106*"[Title/Abstract] OR "bms 936558*"[Title/Abstract] OR "bms 936558*"[Title/Abstract] OR "bms936558*"[Title/Abstract]) OR ("atezolizumab"[Supplementary Concept] OR ("atezolizumab*"[Title/Abstract] OR "anti pdl1*"[Title/Abstract] OR "mpdl3280a*"[Title/Abstract] OR "mpdl 3280a*"[Title/Abstract] OR "tecentriq*"[Title/Abstract] OR "rg7446*"[Title/Abstract] OR "rg 7446*"[Title/Abstract]))) AND (("radiotherapies*"[Title/Abstract] OR "radiation therapy*"[Title/Abstract] OR "radiation therapies*"[Title/Abstract] OR "therapies radiation*"[Title/Abstract] OR "therapy radiation*"[Title/Abstract] OR "radiation treatment*"[Title/Abstract] OR "radiation treatments*"[Title/Abstract] OR "treatment radiation*"[Title/Abstract] OR "radiotherapy targeted*"[Title/Abstract] OR "radiotherapies targeted*"[Title/Abstract] OR "targeted radiotherapies*"[Title/Abstract] OR "targeted radiotherapy*"[Title/Abstract] OR "targeted radiation therapy*"[Title/Abstract] OR (("radiate"[All Fields] OR "radiated"[All Fields] OR "radiates"[All Fields] OR "radiating"[All Fields] OR "Radiation"[MeSH Terms] OR "Radiation"[All Fields] OR "electromagnetic radiation"[MeSH Terms] OR ("electromagnetic"[All Fields] AND "Radiation"[All Fields]) OR "electromagnetic radiation"[All Fields] OR "radiations"[All Fields] OR "radiation s"[All Fields] OR "radiator"[All Fields] OR "radiators"[All Fields]) AND "therapies targeted*"[Title/Abstract]) OR "targeted radiation therapies*"[Title/Abstract] OR (("therapeutics"[MeSH Terms] OR "therapeutics"[All Fields] OR "Therapies"[All Fields] OR "Therapy"[MeSH Subheading] OR "Therapy"[All Fields] OR "therapy s"[All Fields] OR "therapys"[All Fields]) AND "targeted radiation*"[Title/Abstract]) OR "therapy targeted radiation*"[Title/Abstract] OR ("Radiotherapy"[MeSH Terms] OR "Radiotherapy"[All Fields] OR ("Radiation"[All Fields] AND "Therapy"[All Fields] AND "Targeted"[All Fields]) OR "radiation therapy targeted"[All Fields])) AND ("carcinoma non small cell lung*"[Title/Abstract] OR (("carcinoma, non small cell lung"[MeSH Terms] OR ("Carcinoma"[All Fields] AND "Non-Small-Cell"[All Fields] AND "Lung"[All Fields]) OR "non-small-cell lung carcinoma"[All Fields] OR ("Carcinoma"[All Fields] AND "Non"[All Fields] AND "Small"[All Fields] AND "Cell"[All Fields] AND "Lung"[All Fields]) OR "carcinoma non small cell lung"[All Fields]) AND "carcinomas non small cell lung*"[Title/Abstract]) OR "lung carcinoma non small cell*"[Title/Abstract] OR "lung carcinomas non small cell*"[Title/Abstract] OR "non small cell lung carcinomas*"[Title/Abstract] OR "non small cell lung carcinoma*"[Title/Abstract] OR "non small cell lung carcinoma*"[Title/Abstract] OR (("carcinoma, non small cell lung"[MeSH Terms] OR ("Carcinoma"[All Fields] AND "Non-Small-Cell"[All Fields] AND "Lung"[All Fields]) OR "non-small-cell lung carcinoma"[All Fields] OR ("Non"[All Fields] AND "Small"[All Fields] AND "Cell"[All Fields] AND "Lung"[All Fields] AND "Carcinoma"[All Fields]) OR "non small cell lung carcinoma"[All Fields]) AND "nonsmall cell lung cancer*"[Title/Abstract]) OR "carcinoma non small cell lung*"[Title/Abstract] OR "non small cell lung cancer*"[Title/Abstract] OR "carcinomas non small cell lung*"[Title/Abstract] OR "lung carcinoma non small cell*"[Title/Abstract] OR "lung carcinomas non small cell*"[Title/Abstract] OR "non small cell lung carcinomas*"[Title/Abstract] OR "non small cell lung carcinoma*"[Title/Abstract] OR "non small cell lung carcinoma*"[Title/Abstract] OR (("carcinoma, non small cell lung"[MeSH Terms] OR ("Carcinoma"[All Fields] AND "Non-Small-Cell"[All Fields] AND "Lung"[All Fields]) OR "non-small-cell lung carcinoma"[All Fields] OR ("Non"[All Fields] AND "Small"[All Fields] AND "Cell"[All Fields] AND "Lung"[All Fields] AND "Carcinoma"[All Fields]) OR "non small cell lung carcinoma"[All Fields]) AND "nonsmall cell lung cancer*"[Title/Abstract]) OR "carcinoma non small cell lung*"[Title/Abstract] OR ("carcinoma, non small cell lung"[MeSH Terms] OR ("Carcinoma"[All Fields] AND "Non-Small-Cell"[All Fields] AND "Lung"[All Fields]) OR "non-small-cell lung carcinoma"[All Fields] OR ("Non"[All Fields] AND "Small"[All Fields] AND "Cell"[All Fields] AND "Lung"[All Fields] AND "cancer"[All Fields]) OR "non small cell lung cancer"[All Fields]))))

Cochrane

#1 MeSH descriptor: [Carcinoma, Non-Small-Cell Lung] explode all trees 4878

#2 MeSH descriptor: [Radiotherapy] explode all trees 6696

#3 MeSH descriptor: [Immune Checkpoint Inhibitors] explode all trees 91

#4 MeSH descriptor: [Nivolumab] explode all trees 613

#5 MeSH descriptor: [Ipilimumab] explode all trees 278

#6 (durvalumab):ti,ab,kw (Word variations have been searched) 919

#7 (atezolizumab):ti,ab,kw (Word variations have been searched) 1211

#8 (Avelumab):ti,ab,kw (Word variations have been searched) 333

#9 (Pembrolizumab):ti,ab,kw (Word variations have been searched) 2555

#10 (Tremelimumab):ti,ab,kw (Word variations have been searched) 368

#11 (cemiplimab):ti,ab,kw (Word variations have been searched) 81

#12 (camrelizumab):ti,ab,kw (Word variations have been searched) 167

#13 (toripalimab):ti,ab,kw (Word variations have been searched) 97

#14 (sintilimab):ti,ab,kw (Word variations have been searched) 127

#15 (Ipilimumab):ti,ab,kw (Word variations have been searched) 1631

#16 (Nivolumab):ti,ab,kw (Word variations have been searched) 2559

#17 #3 OR #4 OR #5 OR #6 OR #7 OR #8 OR #9 OR #10 OR #11 OR #12 OR #13 OR #14 OR #15 OR #16 7845

#18 (Immune Checkpoint Inhibitors):ti,ab,kw (Word variations have been searched) 1599

#19 (Radiotherapy):ti,ab,kw (Word variations have been searched) 37326

#20 (NSCLC):ti,ab,kw (Word variations have been searched) 10781

#21 #20 OR #1 12519

#22 #19 OR #2 37902

#23 #21 AND #22 AND #17 289

Embase

(('radiotherapy' OR 'radiotherapy'/exp OR radiotherapy) AND ('non small cell lung cancer'/exp OR 'non small cell lung cancer') AND ('pd-l1 inhibitors' OR ('pd l1' AND ('inhibitors' OR 'inhibitors'/exp OR inhibitors)) OR 'pd-1 inhibitors' OR (('pd 1'/exp OR 'pd 1') AND ('inhibitors' OR 'inhibitors'/exp OR inhibitors)) OR 'ctla-4 inhibitors' OR (('ctla 4'/exp OR 'ctla 4') AND ('inhibitors' OR 'inhibitors'/exp OR inhibitors)) OR 'immune checkpoint inhibitor'/exp OR 'immune checkpoint inhibitor' OR 'durvalumab' OR 'durvalumab'/exp OR durvalumab OR 'nivolumab' OR 'nivolumab'/exp OR nivolumab OR 'atezolizumab' OR 'atezolizumab'/exp OR atezolizumab OR 'avelumab' OR 'avelumab'/exp OR avelumab OR 'pembrolizumab' OR 'pembrolizumab'/exp OR pembrolizumab OR 'ipilimumab' OR 'ipilimumab'/exp OR ipilimumab OR 'tremelimumab' OR 'tremelimumab'/exp OR tremelimumab OR 'sintilimab' OR 'sintilimab'/exp OR sintilimab OR 'toripalimab' OR 'toripalimab'/exp OR toripalimab OR 'camrelizumab' OR 'camrelizumab'/exp OR camrelizumab OR 'cemiplimab' OR 'cemiplimab'/exp OR cemiplimab)) AND 'article'/it
