# Supplementary material for: Evaluation safety and efficacy of immune checkpoint blockers (ICB) and radiotherapy combination versus ICB in non‐small cell lung cancer patients with recurrence or metastasis: A systematic review and meta‐analysis
Source: Cancer Med. 2023 Jun 16;12(13):13928–41. doi: 10.1002/cam4.5958 (PMC10358264; doi:10.1002/cam4.5958)
Supplement: Supplementary file 2 — Supplementary Information S2. Table S1. Table S2. Table S3. Table S4. [file CAM4-12-13928-s003.doc]

Table S1: Risk of bias according to the Cochrane Collaboration Risk of Bias Tool for randomized controlled trial studies

| study | Year | Random sequence generation | Allocation concealment | Blinding of participants and personnel | Blinding of outcome assessment | Incomplete outcome data | Selective reporting | Other bias |
| --- | --- | --- | --- | --- | --- | --- | --- | --- |
| Theelen WSME, 2019 | 2019 | low risk | unclear risk | low risk | low risk | low risk | low risk | unclear risk |
| Welsh J, 2020 | 2020 | low risk | low risk | high risk | high risk | low risk | low risk | unclear risk |
| Schoenfeld JD, 2022 | 2022 | low risk | low risk | high risk | high risk | low risk | low risk | unclear risk |

Table S2: Risk of bias according to the Methodological index for non-randomized studies

| study | Clearly stated aim | Inclusion of consecutive patients | Prospective collection of data | Appropriate endpoints | Unbiased assessment of endpoints | Appropriate follow up period | Loss to follow up ≤ 5% | prospective calculation of sample size | total score |
| --- | --- | --- | --- | --- | --- | --- | --- | --- | --- |
| Mattes MD, 2021 | 2 | 2 | 2 | 2 | 0 | 2 | 2 | 2 | 14 |
| Miyamoto S, 2018 | 2 | 2 | 2 | 2 | 0 | 2 | 2 | 2 | 14 |
| Qin A, 2019 | 2 | 2 | 2 | 2 | 1 | 2 | 2 | 2 | 15 |

Table S3: Risk of bias according to Newcastle-Ottawa Scale

|  | Selection | | | | Comparability | Outcome | | |
| --- | --- | --- | --- | --- | --- | --- | --- | --- |
| study | 1 | 2 | 3 | 4 | 1 | 1 | 2 | 3 |
| Bestvina CM, 2022 | * | * | * | * | ** | * | * | * |
| Fiorica F, 2018 | * | * | * | * |  | * | * | * |
| Hubbling HG, 2018 | * | * | * | * | ** | * | * | * |
| Horndalsveen H, 2022 | * | * | * | * | ** | * | * | * |
| Ratbayake G, 2019 | * | * | * | * | ** | * | * | * |
| Samuel E, 2020 | * | * | * | * | ** | * | * | * |
| Shaverdian N, 2017 | * | * | * | * | ** | * | * | * |
| Singh C, 2019 | * | * | * | * | ** | * | * | * |
| Zhang Q, 2020 | * | * | * | * | ** | * | * | * |
| Guo T, 2022 | * | * | * | * | ** | * | * | * |
| Zhou ZC, 2022 | * | * | * | * | ** | * | * | * |
| Yamaguchi O, 2019 | * | * | * | * | ** | * | * | * |

Table S4: Risk of bias according to JBI Critical Appraisal Checklist

| study | 1 | 2 | 3 | 4 | 5 | 6 | 7 | 8 | 9 | 10 | Overall appraisal |
| --- | --- | --- | --- | --- | --- | --- | --- | --- | --- | --- | --- |
| Abdulhaleem M, 2022 | YES | YES | YES | Unclear | YES | YES | YES | YES | YES | YES | Include |
| Amino Y, 2019 | YES | YES | YES | Unclear | YES | YES | YES | YES | YES | YES | Include |
| Bassanelli M, 2022 | YES | YES | YES | Unclear | YES | YES | YES | YES | YES | YES | Include |
| Porte J, 2022 | YES | YES | YES | Unclear | YES | YES | YES | YES | YES | YES | Include |
| Lesueur P, 2018 | YES | YES | YES | YES | YES | YES | YES | NO | YES | YES | Include |
| Schapira E, 2018 | YES | NO | YES | Unclear | YES | YES | YES | YES | YES | YES | Include |
| Tjong MC, 2022 | YES | YES | YES | Unclear | YES | YES | YES | YES | YES | YES | Include |
